# Integrating the ICF and the F-Words Framework to Support Family-Centered Pediatric Care for Children with Neurodevelopmental Disorders: A Narrative Review

**DOI:** 10.3390/children13030354

**Published:** 2026-02-28

**Authors:** Anna Gogola, Rafał Gnat, Aneta Skworc, Jerzy Luszawski, Sławomir Snela

**Affiliations:** 1 Institute of Physiotherapy and Health Sciences, Academy of Physical Education, 40-065 Katowice, Poland; r.gnat@awf.katowice.pl; 2 Developmental Neuro-Motor Stimulation Institute International, 43-600 Jaworzno, Poland; 3 Department of Physiotherapy, Katowice Business University, 40-659 Katowice, Poland; aneta.skworc@akademiagornoslaska.pl; 4 Department of Pediatric Neurosurgery, University Hospital No. 6, Medical University of Silesia, 40-752 Katowice, Poland; 5 Department of Orthopaedics and Traumatology, University of Rzeszów, 35-959 Rzeszów, Poland; ssnela@poczta.onet.pl

**Keywords:** ICF classification, F-words, cerebral palsy, childhood disability, neurodevelopmental disorders

## Abstract

**Highlights:**

**What are the main findings?**

- The ICF and the F-words framework share a strong conceptual alignment with family-centered care, particularly through their emphasis on functioning, participation, and contextual factors meaningful to families.
- Despite their widespread use, both frameworks face persistent challenges related to conceptual clarity, operationalization, outcome measurement, and the consistent involvement of families in clinical decision-making.

**What are the implications of the main findings?**

- Integrating the ICF and the F-words framework using a structured, complementary approach may enhance family-centered care by improving shared decision-making, goal-setting, and communication between clinicians and families.
- Clearer conceptual mapping and practice-oriented guidance are needed to translate family priorities into meaningful, measurable outcomes in pediatric care.

**Abstract:**

**Background**: Family-centered care is a core principle of contemporary pediatric rehabilitation, particularly for children with neurodevelopmental disorders. Conceptual frameworks emphasizing functioning, participation, and contextual factors are essential to operationalize this approach in clinical practice. The International Classification of Functioning, Disability and Health (ICF) and the F-words framework are widely used models aligned with these principles; however, their practical integration remains challenging. **Methods**: A narrative literature review was conducted using PubMed, Scopus, and Web of Science databases to identify English-language publications (2005–2025) addressing the ICF, the F-words framework, and pediatric neurodevelopmental disability. **Results**: Thirty-one publications were included. The findings indicate increasing international adoption of both frameworks in pediatric rehabilitation. However, persistent challenges were identified, including conceptual ambiguity between activity and participation domains, limited operationalization of personal factors, overlap between the ICF and F-words constructs, limited standardization, difficulties in outcome measurement, and inconsistent involvement of families in clinical decision-making. **Conclusions**: The ICF and the F-words framework offer complementary strengths for advancing family-centered pediatric care. A structured dual-layer integration approach may enhance shared decision-making, clarify goal-setting, and improve communication with families. Further methodological refinement and practice-oriented guidance are needed to support consistent clinical implementation.

## 1. Introduction

Since the beginning of the twenty-first century, there has been a dynamic evolution in how human disability is conceptualized [1,2,3,4]. This shift is particularly evident in the treatment and rehabilitation of children with developmental and neurological conditions [1,2,4,5,6]. Disabilities resulting from structural and/or functional disorders of the nervous system are typically complex, often involving the musculoskeletal system and producing cognitive, behavioral, and communication difficulties that can affect quality of life across the lifespan [7].

Broadly, the changing perception of childhood disability reflects a gradual unification of traditional biomedical concepts with modern perspectives that acknowledge the influence of social, environmental, and personal factors on child development. Consequently, contemporary therapeutic approaches aim to enhance both family well-being and the timing and context of professional interventions across multiple domains [6,8].

The traditional approach to childhood disability, dominant in the previous century, was rooted in a biomedical paradigm that emphasized identifying and “repairing” the source of dysfunction and observing resulting health changes [4,9,10,11,12]. While this model remains effective in acute medical treatment, it proves insufficient when addressing chronic developmental conditions. The “repair” approach is limited by diagnostic imprecision, particularly in heterogeneous conditions such as cerebral palsy, the scarcity of evidence-based therapeutic methods, the weak association between impairment-level interventions and real-world functioning, and the difficulty in distinguishing treatment effects from natural developmental or compensatory changes. Accordingly, new perspectives have emerged that promote a broader understanding of disability, recognizing its multidimensional determinants and offering a more comprehensive set of responses [1,2,13,14].

The modern biopsychosocial paradigm, encompassing multiple interacting determinants of child development, presents both opportunities and challenges for clinicians seeking to navigate comprehensive rehabilitation processes effectively. One valuable framework for guiding this complex process is the ICF, introduced by the World Health Organization (WHO) in 2001 as a universal conceptual and classificatory model for describing health and disability [1,7,15,16,17].

Complementary to the ICF, the F-words framework—function, family, fitness, fun, friends, and future—offers a child-friendly model that operationalizes ICF concepts in pediatric practice [18,19,20]. Since its introduction in 2012, this framework has gained international recognition for supporting holistic approaches to childhood disability, informing physical activity and rehabilitation practices, and serving as a tool for assessing quality-of-life outcomes [18]. By translating ICF constructs into accessible and engaging language, the F-words facilitate the implementation of child and family-centered care across diverse rehabilitation contexts. Although widely adopted internationally to guide comprehensive, participation-focused care [5,19,21,22], the F-words framework remains less familiar to some professionals compared to the ICF.

Despite their theoretical and practical potential, both the ICF and the F-words framework remain underutilized in clinical settings. This narrative review aims to introduce and integrate these frameworks, critically analyze their limitations, and propose constructive adjustments to enhance clarity, usability, and application in day-to-day pediatric rehabilitation practice. By doing so, the authors intend to encourage healthcare professionals to adopt these concepts, improving coordination and effectiveness in the management of childhood disability.

Despite their widespread dissemination and conceptual alignment, the practical integration of the ICF and the F-words framework remains inconsistent in everyday clinical settings. Clinicians often encounter conceptual overlap between domains, ambiguity in mapping constructs across frameworks, and limited guidance on how to translate F-words priorities into measurable goals within the ICF structure. As a result, the two frameworks are frequently used in parallel rather than in an integrated manner, reducing their potential to fully support family-centered decision-making. This review addresses this gap by critically analyzing points of conceptual tension and proposing a structured approach to facilitate clearer and more coherent integration in pediatric rehabilitation practice.

The remainder of this paper is organized as follows. Section 2 describes the methodology of the narrative review. Section 3 presents the results. Section 4 discusses the contributions and limitations of the ICF and the F-words framework and proposes directions for their integration. Section 5 and Section 6 outline the limitations of the review and present the conclusions.

## 2. Materials and Methods

### 2.1. Review Design and Research Question

A structured narrative review was conducted to examine the application and integration of the ICF and the F-words framework in paediatric care for children with neurodevelopmental disorders. A narrative approach was selected due to the conceptual and theoretical nature of the research objective, which required synthesis of heterogeneous sources, including empirical studies, reviews, validation reports, clinical guidelines, and policy documents. The aim was not to evaluate intervention effectiveness but to critically analyse conceptual alignment, practical implementation, and integration challenges.

The review was guided by the following research question: How are the ICF and the F-words framework applied in pediatric neurorehabilitation, and what conceptual and practical challenges emerge when integrating these frameworks within family-centred care?

### 2.2. Search Strategy

The literature search was conducted using PubMed, Scopus, and Web of Science databases. Searches were limited to English-language publications published between 2005 and 2025. Keywords were searched within the Title and Abstract fields using Boolean operators and the following search string: (“F-words” AND (“ICF” OR “International Classification of Functioning”) AND (“neurodevelopmental disorders” OR “childhood disability” OR “cerebral palsy”) AND (“review” OR “systematic review” OR “narrative review”)). No age filters were applied beyond the conceptual focus on pediatric populations inherent in the search terms. Database-specific syntax adjustments were made where necessary. The search yielded 64 records (PubMed *n* = 19; Scopus *n* = 18; Web of Science *n* = 27).

### 2.3. Eligibility Criteria

Studies were included if they: (1) addressed the ICF and/or the F-words framework; (2) focused on children or adolescents with neurodevelopmental disorders; (3) discussed conceptual, clinical, or policy applications relevant to family-centered care; or (4) were published in English between 2005 and 2025. Eligible publication types included empirical studies (quantitative and observational), systematic, scoping, and narrative reviews, validation studies, expert reports, and clinical practice guidelines.

Publications were excluded if they: (1) focused exclusively on adult populations; (2) did not reference either the ICF or F-words framework; or (3) were conference abstracts, dissertations, or non-peer-reviewed materials.

### 2.4. Screening Process

**Figure 1:**
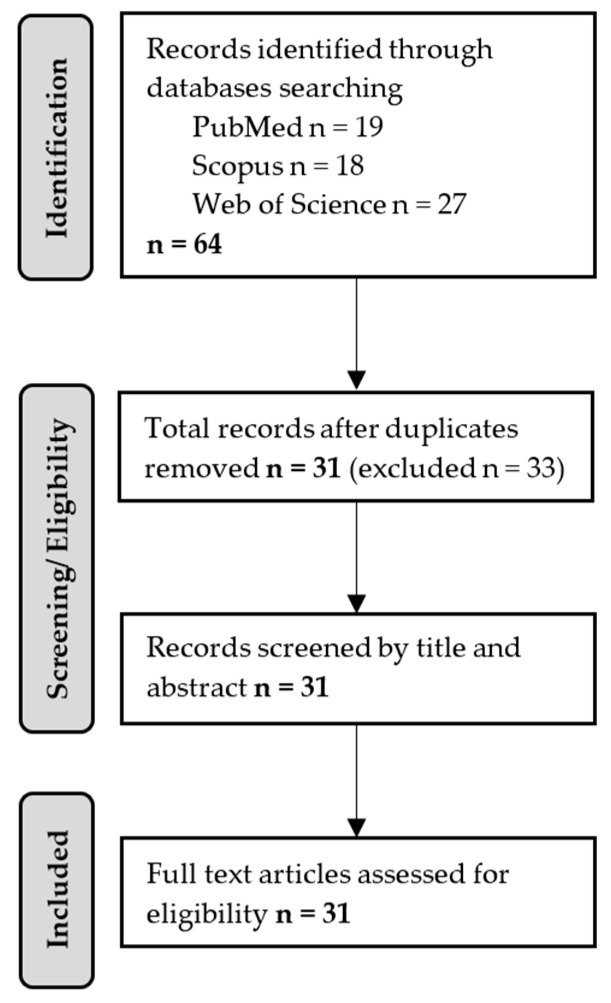
Flow diagram of the study selection process.

After removal of 33 duplicates, 31 records remained. Title and abstract screening confirmed their relevance to the review objectives. No additional exclusions were required at the full-text stage, as all remaining publications met the predefined inclusion criteria (Figure 1). Therefore, 31 publications were included in the final synthesis. Screening and eligibility assessment were conducted independently by two authors, and any uncertainties were resolved through discussion and consensus. All publications remaining after duplicate removal were considered eligible. Thematic saturation was considered achieved, as the available literature did not provide additional conceptual categories or practical insights regarding the integration of the ICF and the F-words frameworks. Given the deliberately narrow conceptual focus of this review and the specificity of the search strategy, the number of retrieved records was limited, and all non-duplicate publications were directly relevant to the research question.

### 2.5. Quality Assessment

As this review focused on conceptual analysis rather than evaluating intervention effectiveness, a formal risk-of-bias or methodological quality assessment was not performed. Nevertheless, attention was given to methodological transparency, relevance, and scholarly credibility during study selection.

## 3. Results

### 3.1. Characteristics of Included Publications

The 31 included publications represent a heterogeneous body of literature comprising conceptual papers, systematic reviews, validation studies, clinical guidelines, policy documents, empirical research, and editorials (Table 1).

A substantial proportion of the literature is conceptual or theoretical in nature, reflecting the ongoing effort to clarify and operationalise the biopsychosocial model of disability and its application in childhood neurodevelopmental conditions. Rather than constituting a single, coherent line of enquiry, the included literature clusters around two primary thematic areas: (1) the conceptual development and theoretical alignment of the ICF and F-words frameworks; and (2) their practical implementation and clinical translation. Two further, secondary themes are also evident: (3) issues of measurement, validation, and operationalization; and (4) evidence syntheses and intervention-oriented perspectives.

### 3.2. Conceptual Alignment and Theoretical Development

A large group of publications elaborates the conceptual foundations of the ICF and the F-words framework, emphasising their shared biopsychosocial orientation and rights-based perspective [18,25,26,27,28,30,31,32,34,40,41,42]. These works collectively argue for a shift from impairment-focused models toward holistic, participation-oriented approaches. The F-words are consistently presented as a pragmatic reframing of the ICF domains, translating abstract classification categories into family-friendly language and practice-oriented constructs [18,40,41].

At the same time, several authors highlight ongoing conceptual ambiguities within the ICF itself, particularly regarding distinctions between activity and participation [25], and the operational interpretation of contextual factors [26,27]. This conceptual complexity partly explains why, despite broad endorsement, integration into daily clinical reasoning remains uneven.

### 3.3. Clinical Implementation and Family-Centered Practice

A second thematic cluster focuses on implementation and knowledge translation [1,5,13,16,19,21,22,29,33]. These studies indicate widespread international dissemination of the F-words framework [19] and growing interest in translating ICF-aligned models into clinical tools and resources [21,29]. Service providers generally report positive attitudes toward the framework, but also describe practical challenges, including time constraints, institutional structures, and uncertainty about how to operationalise holistic principles within discipline-specific interventions [22].

Clinical guidelines and intervention-oriented publications [1,5,13,33] demonstrate partial alignment with ICF domains, yet explicit integration with the F-words framework is often implicit rather than systematically structured. This suggests that while both frameworks are conceptually influential, their combined use is not yet consistently embedded in clinical protocols.

### 3.4. Measurement, Validation, and Operational Challenges

Validation and measurement-focused studies [23,35,36,37,38,39] provide empirical support for ICF-based core sets and functioning measures across various neurodevelopmental conditions. These publications strengthen the structural validity of ICF domains and confirm their applicability across cultural contexts. However, they primarily address classification and measurement rather than practical decision-making processes. Direct empirical operationalisation of the F-words in measurement systems remains comparatively limited, reinforcing the observation that conceptual endorsement exceeds methodological integration.

### 3.5. Evidence Synthesis and Intervention Perspectives

High-level evidence syntheses [2,24] confirm that contemporary intervention research increasingly adopts an ICF-informed lens, particularly in cerebral palsy. Nevertheless, these reviews also reveal that intervention studies frequently prioritise body function outcomes, with comparatively less emphasis on participation and environmental factors. This imbalance reflects a persistent tension between biomedical traditions and holistic frameworks.

## 4. Discussion

The thematic synthesis revealed a consistent pattern: strong conceptual endorsement of the ICF and F-words frameworks coexists with uneven operational integration in clinical practice. The following discussion interprets these findings in relation to structural ambiguities within the ICF model and the need for a more pragmatic, clinically applicable framework.

### 4.1. ICF Framework in Childhood Disability: Contributions and Critical Perspectives

The predominance of conceptual and theoretical publications identified in the Section 3 underscores that much of the discourse around the ICF remains situated at a structural or philosophical level rather than at the level of clinical decision-making. The ICF has profoundly influenced contemporary approaches to neurodevelopmental rehabilitation [7]. By organizing information into the domains of Functioning and Disability (Body Structure and Function; Activity and Participation) and Contextual Factors (Environmental and Personal), it offers a comprehensive biopsychosocial framework that extends beyond the traditional biomedical paradigm [1,2,5,23,24,25,27]. This multidimensional structure enables clinicians and researchers to appreciate the complex interactions between biological, psychological, and social determinants of health, thereby supporting a more holistic conceptualization of childhood disability.

The ICF has also played a transformative role in shaping family-centered and participation-oriented models of care, shifting the focus from ‘fixing’ impairments toward promoting function, participation, and family engagement [28,29]. Its standardized terminology facilitates interdisciplinary communication and comparability of research findings across clinical and research settings [16,23,26]. In addition, the development of ICF Core Sets and Common Data Elements has increased the framework’s applicability in both clinical practice and research [35,36,38,39]. Beyond healthcare, the influence of the ICF extends to policy, aligning with the principles of the United Nations Convention on the Rights of Persons with Disabilities (UNCRPD), which conceptualizes disability as the outcome of interactions between individuals and societal barriers rather than as an intrinsic trait [31,34]. This alignment underscores the value of the ICF as a unifying framework for clinical practice, research, and inclusive policy.

**Figure 2:**
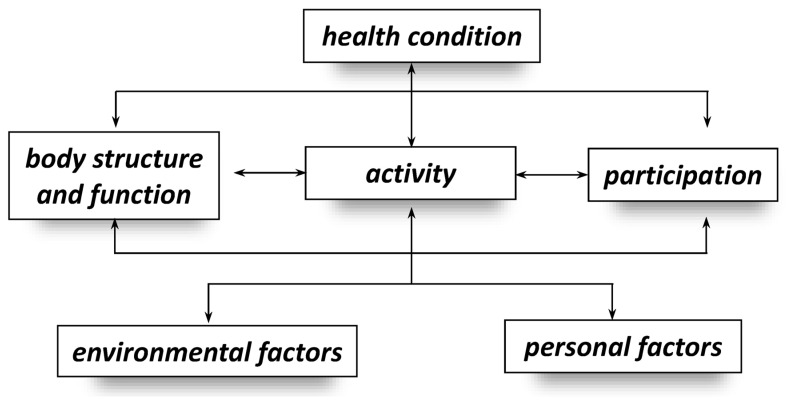
The original diagram of the International Classification of Functioning, Health and Disability components presented by the World Health Organisation [7].

Despite its comprehensiveness and impact, the ICF has been subject to sustained critical analysis. Scholars have noted that the framework lacks clearly articulated theoretical foundations, contains overlapping or partially redundant components, and omits a systematic classification of personal factors [30]. Structural inconsistencies have also been identified between the textual description of the framework and its graphical representation. For example, ‘activity’ and ‘participation’ are presented as separate domains, whereas ‘body structures’ and ‘body functions’ are merged into a single block, as shown in the original WHO diagram (Figure 2). Such inconsistencies may obscure conceptual boundaries and complicate the interpretation of relationships among components, potentially limiting clarity and consistency across clinical and research applications.

These structural ambiguities are not merely theoretical but have tangible consequences for everyday clinical reasoning and goal setting. For example, the conceptual overlap between ‘activity’ and ‘participation’ may lead clinicians to prioritise task execution (e.g., walking a specified distance) while insufficiently addressing participation outcomes such as engagement in peer play or inclusion in school activities. Likewise, because personal factors are not formally coded within the ICF structure, aspects such as motivation, coping style, family expectations, or cultural values may be inconsistently incorporated into goal setting. In practice, these structural ambiguities can inadvertently reinforce impairment-focused planning despite the model’s biopsychosocial intent.

From a conceptual perspective, some authors argue that the ICF may inadvertently overemphasize biological aspects of functioning relative to psychological and social determinants, thereby weakening the balanced biopsychosocial perspective it is intended to uphold [3]. In response to these concerns, Haslam et al. proposed a socio-psycho-bio model that reverses the traditional hierarchy by placing social and group processes at the forefront, psychological processes at an intermediate level, and biological factors as downstream consequences [32]. Within this framework, health phenomena are embedded in social identity, group membership, and collective life, emphasizing that social connectedness and identity processes shape both psychological and biological outcomes.

Additional limitations relate to the restricted distinction between ‘activity’ and ‘participation’ as well as the positioning of personal and environmental factors within the contextual domain [3,25]. In practical terms, challenges have also been reported in implementing the ICF in everyday rehabilitation. The framework’s complexity and the time required for its application—particularly in autism spectrum disorder and other neurodevelopmental conditions—may hinder its consistent use in clinical settings [42]. Moreover, alignment between commonly used clinical assessment tools and ICF categories remains incomplete, leaving some functional domains underrepresented, especially personal factors, which still lack standardized measurement instruments [13,36].

Nevertheless, these limitations do not diminish the conceptual significance of the ICF. Rather, they highlight the need for continued refinement and contextual adaptation to ensure that the framework remains both theoretically robust and practically feasible. When integrated thoughtfully, the ICF continues to serve as a foundational tool for advancing holistic, person-centered, and inclusive approaches to neurodevelopmental rehabilitation.

### 4.2. F-Words in Childhood Disability: Practical Contributions and Conceptual Limitations

The limited number of studies explicitly operationalizing the F-words framework in structured clinical systems suggests that further conceptual development remains ongoing, including proposals to expand the framework.

The F-words framework—comprising function, family, fitness, fun, friends, and future—represents a child-friendly operationalization of the ICF, designed to facilitate holistic and family-centered interventions in pediatric rehabilitation [5,19,21,22,28,33]. Since its introduction in 2012, the framework has gained international recognition, shaping contemporary approaches to childhood disability, informing physical activity and rehabilitation practices, and serving as a tool for evaluating quality-of-life outcomes [19,40]. The F-words emphasize the most meaningful aspects of children’s lives, aligning with the biopsychosocial paradigm by highlighting function, family engagement, physical and mental fitness, joy, peer relationships, and future orientation.

Within this framework, function broadly corresponds to the ICF domains of body structures and functions as well as activities and participation, emphasizing that therapeutic success should be defined not by conformity to normative standards but by meaningful participation in daily tasks and play [18,28]. Family underscores the essential role of caregivers in therapeutic processes, acknowledging that childhood disability affects the well-being of the entire household. Fitness integrates physical and mental health, promoting individualized, ability-based approaches to movement and lifestyle. Fun and friends emphasize joy and social connectedness as intrinsic motivators that enhance engagement in therapy, while future promotes long-term planning aimed at optimizing both present functioning and later quality of life [18].

More recently, freedom has been proposed as an overarching concept linking all six F-words movement, activity and participation (the ability to make choices and engage in diverse experiences), and environmental and personal dimensions (accessibility and autonomy) [3,41]. Central to this notion is the principle of autonomy, which is foundational in both bioethical discourse and UNCRPD [34]. Within this perspective, health can be understood as the freedom to pursue meaningful goals and interests, whereas disability represents a limitation of that freedom. Consequently, interventions should not only aim to reduce impairment but also to enhance participation, dignity, and self-determination. The authors of this paper further extend this view by defining freedom as personal autonomy—the capacity to act and express oneself within one’s own boundaries while minimizing external assistance and dependence on others. Although the proposal to include freedom as an additional F-word has generated conceptual interest, it should be regarded as an emerging extension rather than a formally established component of the framework. Its incorporation reflects ongoing efforts to strengthen the alignment between childhood disability models and rights-based, autonomy-oriented perspectives, but its status within the scientific community remains developmental rather than universally endorsed.

**Figure 3:**
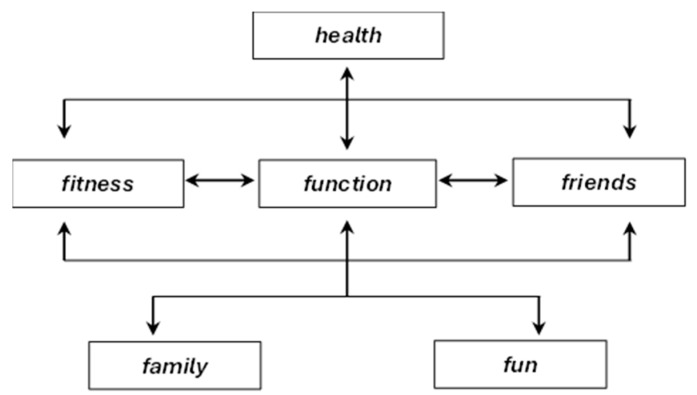
F-words overlaid on the International Classification of Functioning, Health and Disability components diagram [18].

Despite these strengths, integrating the F-words framework with the ICF presents conceptual and practical challenges. Soper et al. argue that the framework remains in an early phase of theoretical development and highlight difficulties in translating its holistic, client-centered principles into consistent, multidisciplinary practice [19,22]. Furthermore, they note that by borrowing from established models such as the ICF, the F-words risk becoming an unquestioned organizing structure that may unintentionally constrain theoretical innovation. Direct attempts to align the F-words with ICF components can create ambiguity. Rosenbaum and Gorter [18], for instance, appear to substitute existing ICF categories (Figure 3) with corresponding F-words, leaving readers uncertain as to whether the two frameworks should be integrated, used alternately, or treated as complementary.

Several F-words overlap with multiple ICF domains—for example, function corresponds simultaneously to body structures and functions and to activities and participation—creating terminological ambiguity and practical challenges [18]. This overlap raises uncertainty as to whether clinicians should replace ICF components with F-words, apply both frameworks in parallel, or treat the F-words as interpretive lenses through which ICF information is understood.

### 4.3. Proposed Solutions

The gaps identified in the Results—particularly the lack of structured integration models—provide the rationale for the pragmatic revisions proposed below.

After highlighting certain points of discussion in the ICF and F-words concepts, it is worth proposing a few practical solutions to existing issues, primarily aimed at reducing ambiguity and eliminating logical gaps. These proposals are presented in the same order as the areas of debate discussed in the previous subsection.

**Figure 4:**
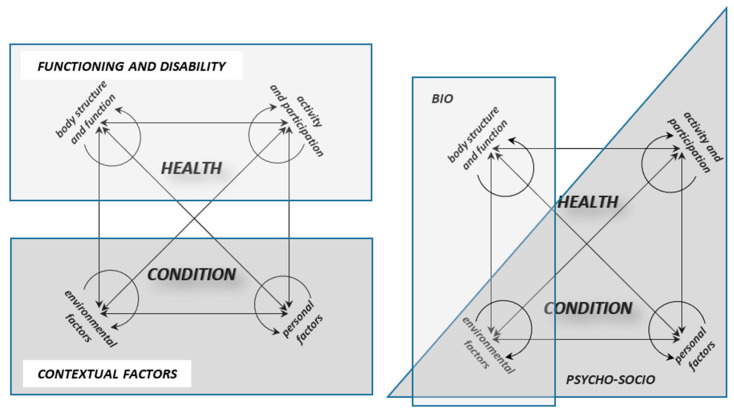
Modified diagram of the International Classification of Functioning, Disability and Health (ICF) components. Additionally, the following are marked: (1) the two main parts of the ICF, i.e., Functioning and Disability and Contextual Factors (on the left), together with their components; (2) ICF components corresponding to the biological and psycho-social medical paradigms (with overlap visible within the environmental factors component). This modified visualization differs from the conventional WHO diagram by presenting symmetrical and reciprocal relationships between all components and positioning health condition as the central outcome of their interaction. The figure aims to clarify conceptual structure and support flexible clinical interpretation of biopsychosocial interactions in pediatric rehabilitation.

First, to address the inconsistencies between the textual description and the graphical representation of the ICF system, we propose a revision of the existing diagram (Figure 2), presented here in Figure 4. In this revised version, the separation of the ‘activity and participation’ component into two subcomponents has been removed, thus simplifying the diagram and aligning it with the official description. Additionally, ‘health condition’ is repositioned as the heading of the framework, which reflects its actual role. This adjustment clarifies that the health condition is not an independent influencing factor, but rather the result of the interactions between the other components. Placing it at the center of the diagram further emphasizes that all ICF components contribute equally to the overall health condition.

The revised diagram is symmetrical and rotation-invariant: regardless of perspective, ‘health condition’ remains the central element, while the surrounding components retain equal distance. Furthermore, all ICF components are interconnected on an “all-to-all” basis (including ‘self-to-self’ feedback loops), with bidirectional arrows to illustrate reciprocal relationships. This layout provides a more faithful visualization of the ICF’s underlying principles, as suggested by Rosenbaum and Gorter, and reflects the model’s dynamic nature.

From a clinical standpoint, this approach offers a more flexible interpretive tool. For example, when deviations are diagnosed in the “body structure and function” component, the clinician can consider both targeted interventions “within” this component and supportive strategies that stem from other domains. In turn, these influences can be traced back to their potential effects on the remaining components, thereby encouraging a broader biopsychosocial perspective when analyzing disability. In Figure 4, the proposed modified diagram is presented twice: on the left side, with a focus on two key ICF sections, and on the right side—highlighting components aligned with the biological and psycho-social paradigms of thinking about health, disease, and disability (the overlap of two paradigms within the ‘environmental factors’ component can be noticed). While the use of such, ‘overlays’‘ is not necessary, they will certainly help the reader systematize their understanding and perspective of the ICF.

**Figure 5:**
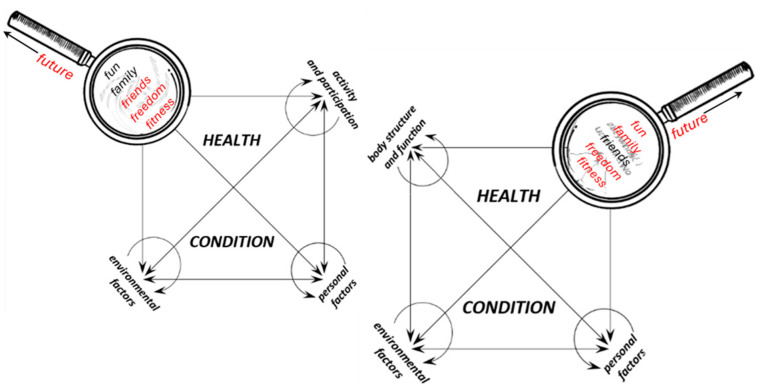
Method of linking the modified diagram of the International Classification of Functioning, Disability and Health (ICF) components with the F-words concept: (1) based on the ICF, the clinician identifies existing deficits; (2) when planning intervention within the body structure and function component, the clinician views this component through a “magnifying glass” containing all F-words; (3) different therapeutic measures illuminate different F-words; (4) preference is given to interventions illuminating the greatest number of F-words; (5) this procedure is repeated across other ICF components. The magnifying glass metaphor represents a pragmatic interpretive lens through which ICF domains are examined in order to align clinical decision-making with family-centered priorities. It does not replace the ICF structure but serves as a conceptual aid to facilitate integration.

With regard to the F-words, a clearer strategy for integration into the ICF framework is required. Instead of simply substituting ICF components with equivalent F-words, we suggest adopting a dual-layer mapping system. In this approach, the F-words act as a pragmatic overlay that translates the abstract ICF categories into family-centered and practice-friendly concepts. For instance, function could serve as a bridge across both ‘body structures and functions’ and ‘activities and participation’ while friends could be directly mapped onto the ‘participation’ domain. Similarly, family and fun could be anchored within the contextual factors, thereby strengthening the psychosocial dimension of the model (Figure 5). The final F-word, future, has been placed on the handle of the magnifying glass as a general guiding principle for the therapeutic process. It ought to be firmly rooted in the ‘now’ while focusing on the ‘later’ aiming—within the existing constraints—to achieve the highest possible level of quality of life and personal happiness [41].

Such mapping would not replace the ICF but would allow both systems to operate in parallel: the ICF as a standardized classification tool, and the F-words as an accessible framework for clinical practice and communication with families. This dual structure could minimize conceptual overlaps, reduce terminological confusion, and enhance the practical utility of both frameworks. Ultimately, the proposed solution aims to preserve the scientific robustness of the ICF while leveraging the intuitive and motivational appeal of the F-words. At this stage, however, the revised diagram and the proposed dual-layer integration model have not yet undergone formal empirical evaluation with clinical professionals. They are presented as theoretically grounded, practice-oriented syntheses derived from the thematic analysis conducted in this review. Future research should assess their usability, clarity, and impact on clinical reasoning through structured implementation and feasibility studies.

## 5. Limitations

This review has several limitations that should be acknowledged. First, as a narrative review, it does not follow the methodological rigor of a systematic review or meta-analysis. Although a structured search strategy and transparent screening process were applied, the absence of a formal quality appraisal of included studies may limit the strength of the conclusions drawn.

Second, the deliberately narrow conceptual focus of the review and the specificity of the search string resulted in a relatively small body of literature. While all records remaining after duplicate removal were directly relevant to the research question, the limited number of publications may restrict the breadth of perspectives represented.

Importantly, the predominance of conceptual, theoretical, and narrative publications among the included articles, with comparatively fewer empirical studies, constitutes an additional limitation. This distribution reflects the current stage of development of the field but conditions the possibility of extracting robust, practice-based conclusions. As a result, some of the proposed solutions presented in this paper remain conceptually grounded and require empirical validation in real-world clinical contexts.

Finally, the proposed revisions to the ICF diagram and the dual-layer integration model have not yet been formally tested with clinical professionals. Future research should evaluate their feasibility, clarity, and applicability in multidisciplinary rehabilitation settings.

## 6. Conclusions

This narrative review examined how the ICF and the F-words framework are applied in paediatric neurorehabilitation and identified conceptual and practical challenges in their integration within family-centred care. Although both frameworks are strongly endorsed and aligned with the biopsychosocial paradigm, their operational integration in clinical practice remains inconsistent and insufficiently structured.

The literature highlights structural ambiguities within the ICF (particularly regarding activity and participation and the representation of personal factors) and conceptual overlaps when F-words are directly mapped onto ICF domains. Moreover, the predominance of conceptual rather than empirical studies limits the availability of evidence-based integration models.

The principal contribution of this review is the synthesis of these tensions and the proposal of a pragmatic solution: a revised visualization of the ICF and a dual-layer integration model in which the F-words function as a clinical interpretive overlay rather than as substitutes for ICF components. This approach maintains the classificatory integrity of the ICF while enhancing its accessibility in family-centred paediatric rehabilitation.

Clinically, the key message is that the ICF and the F-words are best understood as complementary tools: the ICF provides structural coherence, while the F-words offer a practice-oriented lens that facilitates communication and goal-setting. Future research should move beyond conceptual debate toward empirical validation of structured integration models, including evaluation of their feasibility, interdisciplinary acceptability, and measurable impact on clinical reasoning, family engagement, and rehabilitation outcomes.

## Figures and Tables

**Table 1:** Distribution of included articles by publication type, key authors, and number of publications.

Publication Type	Key Authors	Number of Articles
Non-experimental quantitative research	Leonardi et al. (2022) [23]	1
Systematic reviews	Novak et al. (2020) [24]; Damiano et al. (2021) [2];	2
Review articles (scoping, narrative)	Badley (2008) [25]; Rosenbaum & Gorter (2012) [18]; Madden & Bundy, (2019) [26]; Cieza & Kostansjek (2021) [27]; Rosenbaum & Novak-Pavlic (2021) [28]; Shanmugarajah et al. (2021) [29]; Sulaiman et al. (2021) [30]; Longo et al. (2023) [5]; Chagas et al. (2023) [1]; Gogola & Gnat (2025) [13];	10
Expert reports	WHO (2011) [31]	1
Theoretical/conceptual article	Haslam et al. (2019) [32];	1
Clinical practice guideline	Jackman et al. (2022) [33];	1
International legal treaty	United Nations (2006) [34];	1
Validation studies/reports	Cross et al. (2018) [21]; Liao et al. (2020) [35]; D’Arcy et al. (2022) [36]; Schiariti et al. (2024) [37]; Hayden-Evans et al. (2025) [38];	5
Descriptive comparative study	Schiariti et al. (2018) [39]	1
Research studies	Tempest & Jefferson (2015) [16]; Soper et al. (2019, 2020) [19,22];	4
Editorials	Leite et al., (2021) [40]; Dan (2023, 2024) [3,41]; Bölte (2023) [42].	4
Total		31

## Data Availability

No new data were created or analyzed in this study. Data sharing is not applicable to this article.

## Abbreviations

ICF	International Classification of Functioning, Disability and Health
UNCRPD	United Nations Convention on the Rights of Persons with Disabilities
WHO	World Health Organization

## References

[1] Chagas P.S.C., Magalhães E.D.D., Sousa Junior R.R., Romeros A.C.S.F., Palisano R.J., Leite H.R., Rosenbaum P. (2023). Development of Children, Adolescents, and Young Adults with Cerebral Palsy According to the ICF: A Scoping Review. Dev. Med. Child Neurol..

[2] Damiano D.L., Longo E., de Campos A.C., Forssberg H., Rauch A. (2021). Systematic Review of Clinical Guidelines Related to Care of Individuals with Cerebral Palsy as Part of the World Health Organization Efforts to Develop a Global Package of Interventions for Rehabilitation. Arch. Phys. Med. Rehabil..

[3] Dan B. (2024). The ICF as a Socio-Psycho-Biological Model for the Full Participation of Disabled Individuals. Dev. Med. Child Neurol..

[4] Howard J.J., Willoughby K., Thomason P., Shore B.J., Graham K., Rutz E. (2023). Hip Surveillance and Management of Hip Displacement in Children with Cerebral Palsy: Clinical and Ethical Dilemmas. J. Clin. Med..

[5] Longo E., Monteiro R., Hidalgo-Robles Á., Paleg G., Shrader C., De Campos A.C. (2023). Assigning F-Words as Ingredients of Interventions for Children with Cerebral Palsy Functioning at GMFCS IV and V: A Scoping Review Protocol. Front. Rehabil. Sci..

[6] NSW Health (2018). Management of Cerebral Palsy in Children: A Guide for Allied Health Professionals.

[7] World Health Organization (2001). International Classification of Functioning, Disability and Health.

[8] National Institute for Health and Care Excellence (NICE) (2016). Spasticity in Under 19s: Management.

[9] Miller S.D., Mayson T.A., Mulpuri K., O’Donnell M.E. (2020). Developing a Province-Wide Hip Surveillance Program for Children with Cerebral Palsy: From Evidence to Consensus to Program Implementation: A Mini-Review. J. Pediatr. Orthop. B.

[10] te Velde A., Morgan C., Finch-Edmondson M., McNamara L., McNamara M., Paton M.C.B., Stanton E., Webb A., Badawi N., Novak I. (2022). Neurodevelopmental Therapy for Cerebral Palsy: A Meta-Analysis. Pediatrics.

[11] Rutka M., Myśliwiec A., Wolny T., Gogola A., Linek P. (2021). Influence of Chest and Diaphragm Manual Therapy on the Spirometry Parameters in Patients with Cerebral Palsy: A Pilot Study. BioMed Res. Int..

[12] Biały M., Adamczyk W.M., Stranc T., Gogola A., Gnat R. (2025). The Association between Pelvic Asymmetry and Lateral Abdominal Muscle Activity in a Healthy Population. J. Hum. Kinet..

[13] Gogola A., Gnat R. (2025). Evidence-Based Classification, Assessment, and Management of Pain in Children with Cerebral Palsy: A Structured Review. Healthcare.

[14] Myśliwiec A., Saulicz E., Kuszewski M., Wolny T., Knapik A., Gogola A. (2015). Self-Evaluation of the Preparation of Physicians and Physiotherapists to Provide Medical Services to People with Intellectual Disability. J. Intellect. Dev. Disabil..

[15] Rosenbaum P. (2016). Changing the Discourse: We All Must Be Knowledge Brokers. Dev. Med. Child Neurol..

[16] Tempest S., Jefferson R. (2015). Engaging with Clinicians to Implement and Evaluate the ICF in Neurorehabilitation Practice. NeuroRehabilitation.

[17] Gogola A., Gnat R., Snela S., Luszawski J., Filip D., Muzalewski A., Paulitsch F. (2025). Effects of Interdisciplinary Therapy in A Patient with Severe Dystonic Cerebral Palsy: A 12-Year Follow-up Case Report. Int. J. Spec. Edu..

[18] Rosenbaum P., Gorter J.W. (2012). The “F-Words” in Childhood Disability: I Swear This Is How We Should Think. Child Care Health Dev..

[19] Soper A.K., Cross A., Rosenbaum P., Gorter J.W. (2019). Exploring the International Uptake of the “F-Words in Childhood Disability”: A Citation Analysis. Child Care Health Dev..

[20] Gogola A., Gnat R. (2025). Effects of 12-Week Infant Shantala Massage Program on Maternal Emotional Well-Being Following First-Time Birth. Healthcare.

[21] Cross A., Rosenbaum P., Grahovac D., Brocklehurst J., Kay D., Baptiste S., Gorter J.W. (2018). A Web-Based Knowledge Translation Resource for Families and Service Providers (the “f-Words” in Childhood Disability Knowledge Hub): Developmental and Pilot Evaluation Study. JMIR Rehabil. Assist. Technol..

[22] Soper A.K., Cross A., Rosenbaum P., Gorter J.W. (2020). Service Providers’ Perspectives on Using the ‘F-Words in Childhood Disability’: An International Survey. Phys. Occup. Ther. Pediatr..

[23] Leonardi M., Lee H., Kostanjsek N., Fornari A., Raggi A., Martinuzzi A., Yáñez M., Almborg A.-H., Fresk M., Besstrashnova Y. (2022). 20 Years of ICF—Uses and Applications around the World. Int. J. Environ. Res. Public Health.

[24] Novak I., Morgan C., Fahey M., Finch-Edmondson M., Galea C., Hines A., Langdon K., Namara M.M., Paton M.C., Popat H. (2020). State of the Evidence Traffic Lights 2019: Systematic Review of Interventions for Preventing and Treating Children with Cerebral Palsy. Curr. Neurol. Neurosci. Rep..

[25] Badley E.M. (2008). Enhancing the Conceptual Clarity of the Activity and Participation Components of the International Classification of Functioning, Disability, and Health. Soc. Sci. Med..

[26] Madden R.H., Bundy A. (2019). The ICF Has Made a Difference to Functioning and Disability Measurement and Statistics. Disabil. Rehabil..

[27] Cieza A., Kostansjek N. (2021). The International Classification of Functioning, Disability and Health: The First 20 Years. Dev. Med. Child Neurol..

[28] Rosenbaum P.L., Novak-Pavlic M. (2021). Parenting a Child with a Neurodevelopmental Disorder. Curr. Dev. Disord. Rep..

[29] Shanmugarajah K., Rosenbaum P., Zubairi M., Di Rezze B. (2021). A Narrative Review of Function-Focused Measures for Children with Neurodevelopmental Disorders. Front. Rehabil. Sci..

[30] Sulaiman S.K., Mohammad A.H., Ibrahim A.A., Abdu S.I., Kaka B. (2021). Revisiting the International Classification of Functioning, Disability and Health, a Comprehensive Model for Exploring Disablement in Low and Middle-Income Countries: A Narrative Overview. Iran. Rehabil. J..

[31] World Health Organization (2011). World Report on Disability.

[32] Haslam S.A., Haslam C., Jetten J., Cruwys T., Bentley S. (2019). Group Life Shapes the Psychology and Biology of Health: The Case for a Sociopsychobio Model. Soc. Personal. Psychol. Compass.

[33] Jackman M., Sakzewski L., Morgan C., Boyd R.N., Brennan S.E., Langdon K., Toovey R.A.M., Greaves S., Thorley M., Novak I. (2022). Interventions to Improve Physical Function for Children and Young People with Cerebral Palsy: International Clinical Practice Guideline. Dev. Med. Child Neurol..

[34] United Nations (2006). Convention on the Rights of Persons with Disabilities. Treaty Series.

[35] Liao H.F., Hwang A.W., Schiariti V., Yen C.F., Chi W.C., Liou T.H., Hung H.C., Hsieh Y.H. (2020). Validating the ICF Core Set for Cerebral Palsy Using a National Disability Sample in Taiwan. Disabil. Rehabil..

[36] D’Arcy E., Wallace K., Chamberlain A., Evans K., Milbourn B., Bölte S., Whitehouse A.J.O., Girdler S. (2022). Content Validation of Common Measures of Functioning for Young Children against the International Classification of Functioning, Disability and Health and Code and Core Sets Relevant to Neurodevelopmental Conditions. Autism.

[37] Schiariti V., Shierk A., Stashinko E.E., Sukal-Moulton T., Feldman R.S., Aman C., Mendoza-Puccini M.C., Brandenburg J.E., National Institute of Neurological Disorders and Stroke Cerebral Palsy Common Data Elements Oversight Committee (2024). Cerebral Palsy Pain Instruments: Recommended Tools for Clinical Research Studies by the National Institute of Neurological Disorders and Stroke Cerebral Palsy Common Data Elements Project. Dev. Med. Child Neurol..

[38] Hayden-Evans M., Evans K., Milbourn B., D’Arcy E., Chamberlain A., Afsharnejad B., Whitehouse A., Bölte S., Girdler S. (2025). Validating the International Classification of Functioning, Disability and Health Core Sets for Autism in a Sample of Australian School-Aged Children on the Spectrum. J. Autism Dev. Disord..

[39] Schiariti V., Mahdi S., Bölte S. (2018). International Classification of Functioning, Disability and Health Core Sets for Cerebral Palsy, Autism Spectrum Disorder, and Attention-Deficit–Hyperactivity Disorder. Dev. Med. Child Neurol..

[40] Leite H.R., de Carvalho Chagas P.S., Rosenbaum P. (2021). Childhood Disability: Can People Implement the F-Words in Low and Middle-Income Countries—And How?. Braz. J. Phys. Ther..

[41] Dan B. (2023). Freedom: An F-Word for Functioning, Disability, and Health. Dev. Med. Child Neurol..

[42] Bölte S. (2023). A More Holistic Approach to Autism Using the International Classification of Functioning: The Why, What, and How of Functioning. Autism.

